# Association between triglyceride to high-density lipoprotein cholesterol ratio and new-onset diabetes mellitus

**DOI:** 10.20945/2359-4292-2026-0079

**Published:** 2026-07-22

**Authors:** Chunhui Yin, Qi Qi, Xinyu Wu, Lei Li, Yue Jiang, Jing Yu, Yun Zhang, Quanle Han, Shouling Wu, Kangbo Li

**Affiliations:** 1 Department of Cardiology, Tangshan Gongren Hospital, Tangshan, China; 2 Hebei Medical University, Shijiazhuang, China; 3 Catheterization Unit, Tangshan Gongren Hospital, Tangshan, China; 4 Department of Geriatrics, Tangshan Gongren Hospital, Tangshan, China; 5 Department of Cardiology, Kailuan General Hospital, Tangshan, China; 6 School of Clinical Medicine, North China University of Science and Technology, Tangshan, China

**Keywords:** Triglyceride to high-density lipoprotein cholesterol ratio, insulin resistance, diabetes mellitus

## Abstract

**Objective:**

To examine the association between triglyceride to high-density lipoprotein cholesterol (TG/HDL-C) ratio and the onset of diabetes mellitus (DM) within an ongoing prospective cohort in China.

**Subjects and methods:**

Participants were categorized into four groups according to their TG/HDL-C ratio quartiles. Kaplan-Meier estimator determined the cumulative incidence during follow-up and generated time-to-event curves. Additionally, a Cox proportional hazards regression analysis assessed the hazard ratios (HRs) and their corresponding 95% confidence intervals (CIs) for new-onset DM. A sensitivity analysis was also performed to mitigate the possible effects of reverse causation.

**Results:**

During a median follow-up duration of 13.67 years, 38,210 individuals developed DM. Kaplan-Meier curves revealed that the cumulative DM incidence across quartiles 1 to 4 was 40.95%, 42.04%, 41.42%, and 44.38%, respectively. The risk of developing DM increased over time according to the baseline TG/HDL-C ratio quartiles. After adjusting for potential confounding variables, the HRs reached 1.045 (95% CI, 1.015-1.076), 1.026 (95% CI, 0.996-1.058), and 1.095 (95% CI, 1.058-1.133) for quartiles 2, 3, and 4, respectively (P for trend < 0.001). These findings were consistent in sensitivity analyses, with HRs of 1.045 (95% CI, 1.016-1.075), 1.026 (95% CI, 0.996-1.058), and 1.095 (95% CI, 1.058-1.133) for quartiles 2, 3, and 4, respectively (P for trend < 0.0001).

**Conclusion:**

TG/HDL-C ratio is significantly and positively associated with new-onset DM.

## INTRODUCTION

A 2022 report informed that 14% of adults aged 18 and older were living with diabetes mellitus (DM), a significant global increase from 7% in 1990. Additionally, DM was directly responsible for 1.6 million deaths, of which 47% occurred in individuals under 70 (^1^). Most recent data indicates that DM prevalence in China stands at 11.9% (^2^), surpassing the 10.5% global average (^3^). The pivotal pathogenic component of DM is insulin resistance (IR), which is characterized by lack of response to circulating insulin levels leading to fasting hyperglycemia. Research has shown that IR is linked to alterations in lipid and lipoprotein metabolism, resulting in the development of dyslipidemia and the characteristic lipid triad (^4^), a clinical picture marked by an increase in triglyceride-rich particles, a decrease in high-density lipoprotein cholesterol particles, and an increase in low-density lipoprotein cholesterol particles (^5^). Recent research indicated a strong association between the triglyceride to high-density lipoprotein cholesterol (TG/HDL-C) ratio and IR (^6^,^7^). However, relevant studies investigating the relationship between the TG/HDL-C ratio and DM risk are scarce. Thus, our objective is to examine the association between the TG/HDL-C ratio and DM onset within an ongoing prospective cohort study based in China.

## SUBJECTS AND METHODS

### Study population

Data were obtained from the Kailuan study (registration number in the Chinese clinical trial registry: ChiCTR-TNRC-11001489) (Registration Date: 2011-08-24), a prospective cohort investigation conducted within the Kailuan community in Tangshan, China. Briefly, the study involved 101,510 participants, aged between 18 and 98 years, who completed the initial survey from June 2006 to October 2007. Participants underwent assessments through questionnaires, physical examinations, and laboratory tests. Subsequently, all participants were followed up biennially to update the aforementioned information. In the present study, we excluded 9,456 individuals with a history of myocardial infarction, 327 individuals with a history of cancer, and 1,281 individuals with incomplete baseline data regarding triglycerides (TG) and high-density lipoprotein cholesterol (HDL-C) levels. Thus, a total of 90,446 participants were included in the current analysis.

### Data collection and grouping

**Figure 1:**
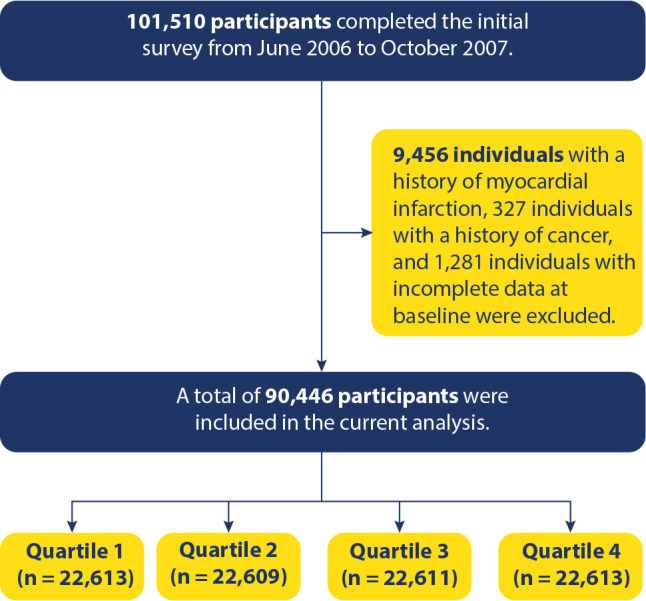
Study flowchart.

Baseline characteristics were collected by trained professionals using a questionnaire that has been published previously (^8^). Participants’ smoking and drinking statuses were categorized as current, former, or never users. Regular physical activity was defined as engaging in exercise at least four times per week, minimum duration of 20 minutes per session. Educational level was classified into five categories: illiterate, primary education, lower secondary education, upper secondary education, and higher education. Various biochemical indices, including total cholesterol (TC), TG, HDL-C, low-density lipoprotein cholesterol (LDL-C), fasting plasma glucose (FPG), serum creatinine (Scr), and high-sensitivity C-reactive protein (hs-CRP), were assessed. TG/HDL-C ratio was calculated by dividing the TG serum concentration by that of HDL-C. Based on the TG/HDL-C ratio quartiles, participants were divided into four groups: quartile 1 (TG/HDL-C ratio ≤ 0.56), quartile 2 (0.56 < TG/HDL-C ratio ≤ 0.83), quartile 3 (0.83 < TG/HDL-C ratio ≤ 1.32), and quartile 4 (TG/HDL-C ratio > 1.32) (**Figure 1**).

### DM diagnosis and follow-up

The study endpoint was defined as a new DM diagnosis. The diagnostic criteria employed aligned with the latest diabetes diagnosis criteria (^9^). Study participants were monitored from the initial assessment until either a new diabetes diagnosis, date of the final physical examination, or death, depending on which event occurred first.

### Statistical analysis

All statistical analyses were conducted on SAS version 9.4. Continuous variables are presented as mean ± standard deviation when they conform to a normal distribution; otherwise, they are reported as median and interquartile range. Categorical variables are shown as numbers and percentages. Differences among the Kaplan-Meier estimates for groups were assessed using the log-rank test. Cox proportional hazards regression analysis was performed to estimate the hazard ratios (HRs) along with their 95% confidence intervals (CIs) for occurrence of the primary endpoint. A sensitivity analysis was conducted to eliminate the potential influence of reverse causation.

## RESULTS

### Baseline characteristics

Table 1 presents the baseline characteristics for the 90,446 study participants. Mean age was 51.36 years, with a standard deviation of 12.74 years, and 72,014 (79.62%) participants were male. Patients with a higher TG/HDL-C ratio exhibited elevated levels of body mass index, Scr, hs-CRP, blood pressure, and FPG. Hypertension, hyperlipemia, and chronic kidney disease showed greater prevalence in the sample, as did current smokers and drinkers. Notably, these participants were more likely to use hypotensive drugs and hypolipidemic drugs.

**Table 1:** Baseline characteristics

Variables	Total (n = 90,446)	Quartile 1 (n = 22,613)	Quartile 2 (n = 22,609)	Quartile 3 (n = 22,611)	Quartile 4 (n = 22,613)	*P* Value
Age, years	51.36 ± 12.74	51.39 ± 13.44	51.33 ± 12.98	51.79 ± 12.58	50.94 ± 11.90	<0.0001
Male, n (%)	72,014 (79.62)	16,756 (74.10)	17,769 (78.59)	18,299 (80.93)	19,190 (84.86)	<0.0001
BMI, kg/m^2^	24.93 ± 3.48	23.40 ± 3.23	24.54 ± 3.29	25.46 ± 3.34	26.33 ± 3.34	<0.0001
TC, mmol/L	4.92 ± 1.12	4.83 ± 0.97	4.94 ±1.00	5.04 ± 1.02	4.88 ± 1.43	<0.0001
TG, mmol/L	1.24 (0.88-1.87)	0.70 (0.57-0.85)	1.07 (0.92-1.23)	1.47 (1.25-1.73)	2.63 (2.04-3.74)	<0.0001
HDL-C, mmol/L	1.55 ± 0.40	1.78 ± 0.44	1.58 ± 0.34	1.46 ± 0.33	1.36 ± 0.35	<0.0001
LDL-C, mmol/L	2.34 ± 0.90	2.20 ± 0.92	2.39 ± 0.85	2.43 ± 0.89	2.33 ± 0.93	<0.0001
TG/HDL-C ratio	1.14 ± 1.33	0.41 ± 0.10	0.69 ± 0.08	1.04 ± 0.14	2.40 ± 2.17	<0.0001
Scr, umol/L	91.69 ± 29.41	86.94 ± 23.54	92.72 ± 27.89	92.08 ± 29.83	95.02 ± 34.71	<0.0001
hs-CRP, mg/L	0.80 (0.30-2.23)	0.62 (0.22-2.01)	0.73 (0.29-2.04)	0.88 (0.33-2.30)	1.00 (0.40-2.59)	<0.0001
SBP, mmHg	130.18 ± 20.79	126.28 ± 20.74	129.46 ± 20.66	131.41 ± 20.63	133.58 ± 20.42	<0.0001
DBP, mmHg	83.19 ± 11.71	80.45 ± 11.34	82.65 ± 11.42	83.97 ± 11.65	85.71 ± 11.79	<0.0001
FBG, mmol/L	5.08 ± 0.70	4.96 ± 0.67	5.06 ± 0.67	5.12 ± 0.70	5.17 ± 0.72	<0.0001
Hypertension, n (%)	38,409 (42.47)	7,420 (32.81)	9,213 (40.75)	10,303 (45.57)	11,473 (50.74)	<0.0001
Hyperlipemia, n (%)	52,581 (58.14)	8,016 (35.45)	9,066 (40.10)	13,484 (59.63)	22,015 (97.36)	<0.0001
Chronic kidney disease, n (%)	12,335 (13.64)	2,059 (9.11)	3,545 (15.68)	3,129 (13.84)	3,602 (15.63)	<0.0001
Hypotensive drugs, n (%)	6,682 (7.39)	1,064 (4.71)	1,395 (6.17)	1,861 (8.23)	2,362 (10.45)	<0.0001
Hypolipidemic drugs, n (%)	5,889 (6.51)	925 (4.09)	1,262 (5.58)	1,615 (7.14)	2,087 (9.23)	<0.0001
Income >800 yuan/month, n (%)	12,974 (14.34)	3,503 (15.49)	2,925 (12.94)	3,191 (14.11)	3,355 (14.84)	<0.0001
High education background, n (%)	18,615 (20.58)	5,176 (22.89)	4,241 (18.76)	4,481 (19.82)	4,717 (20.86)	<0.0001
Regular physical exercise, n (%)	82,500 (91.21)	20,777 (91.88)	20,749 (91.77)	20,519 (90.75)	20,455 (90.46)	<0.0001
Current smoker, n (%)	31,367 (34.68)	7,452 (32.95)	7,121 (31.50)	7,918 (35.02)	8,876 (39.25)	<0.0001
Current drinker, n (%)	34,195 (37.81)	8,661 (38.30)	7,621 (33.71)	8,426 (37.27)	9,487 (41.95)	<0.0001

Abbreviations: BMI: body mass index; TC: total cholesterol; TG: triglycerides; HDL-C: high density lipoprotein; LDL-C: low density lipoprotein; TG/HDL-C ratio: triglyceride to high-density lipoprotein cholesterol ratio; FBG: fasting blood glucose; Scr: serum creatinine; hs-CRP: high sensitivity C-reactive protein, SBP: systolic blood pressure; DBP: diastolic blood pressure.

### Risk of new-onset DM

**Figure 2:**
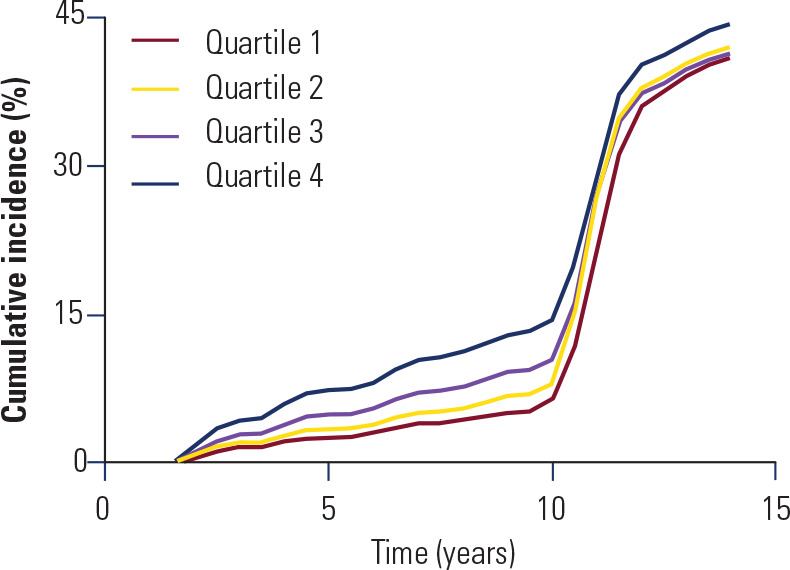
Cumulative incidence of new-onset DM in different quartiles.

During a median follow-up period of 13.67 years, 38,210 cases of new-onset DM were reported. Kaplan-Meier curves indicated that the cumulative DM incidence in quartile 1-4 was 40.95%, 42.04%, 41.42%, and 44.38%, respectively (**Figure 2**). Additionally, the DM incidence rates in quartile 1-4 were 32.71, 34.10, 33.91, and 37.30 per 1000 person-years (**Table 2**).

**Table 2:** Risk and hazard ratios for new-onset DM

	Case/Total	Incidence rate, per 1000 person-years (%)	Model 1	Model 2	Model 3
Quartile 1	9,271/22,613	32.71	1	1	1
Quartile 2	9,521/22,609	34.10	1.072 (^1^,042-^1^,103)	1.104 (^1^,073-^1^,136)	1.058 (^1^,028-^1^,089)
Quartile 3	9,380/22,611	33.91	1.061 (^1^,031-^1^,092)	1.114 (^1^,082-^1^,146)	1.043 (^1^,012-^1^,075)
Quartile 4	10,038/22,613	37.30	1.177 (^1^,145-^1^,211)	1.219 (^1^,185-^1^,255)	1.118 (^1^,080-^1^,156)
*P* for trend			<0.001	<0.001	<0.001

**Model 1** was unadjusted. **Model 2** was adjusted for age and sex. **Model 3** was adjusted for age, sex, body mass index, total cholesterol, low-density lipoprotein cholesterol, serum creatinine, high sensitivity C-reactive protein, systolic blood pressure, diastolic blood pressure, history of hypertension, history of hyperlipemia, history of chronic kidney disease, hypotensive drugs usage, hypolipidemic drugs usage, income level, education level, physical activity, status of smoking, and status of drinking.

### Hazard ratios and 95% confidence intervals

Risk of developing DM increased over time by baseline TG/HDL-C ratio quartiles in univariate model, with hazard ratios (HRs) of 1.072 (95% CI: 1.042-1.103), 1.061 (95% CI: 1.031-1.092), and 1.177 (95% CI: 1.145-1.211) for quartiles 2, 3 and 4, respectively (*P* for trend < 0.001). After adjustment for potential confounding factors, the HRs reached 1.058 (95% CI: 1.028--.089), 1.043 (95% CI: 1.012-1.075), and 1.118 (95% CI: 1.080-1.156) for quartiles 2, 3 and 4, respectively (*P* for trend < 0.001) (**Table 2**). The results remained consistent across three sensitivity analyses (**Table 3**).

**Table 3:** Sensitivity analysis

	Model 1	Model 2	Model 3
Analysis 1			
Quartile 1	1	1	1
Quartile 2	1.066 (1.036-1.098)	1.101 (1.069-1.133)	1.058 (1.028-1.090)
Quartile 3	1.047 (1.017-1.078)	1.102 (1.070-1.135)	1.038 (1.007-1.070)
Quartile 4	1.148 (1.115-1.181)	1.193 (1.159-1.228)	1.102 (1.065-1.141)
*P* for trend	<0.001	<0.001	<0.001
Analysis 2			
Quartile 1	1	1	1
Quartile 2	1.073 (1.042-1.105)	1.105 (1.072-1.138)	1.044 (1.013-1.076)
Quartile 3	1.065 (1.034-1.097)	1.115 (1.082-1.149)	1.027 (0.995-1.059)
Quartile 4	1.173 (1.138-1.208)	1.213 (1.177-1.249)	1.085 (1.047-1.124)
*P* for trend	<0.001	<0.001	<0.001
Analysis 3			
Quartile 1	1	1	1
Quartile 2	1.071 (1.042-1.102)	1.104 (1.073-1.135)	1.058 (1.028-1.089)
Quartile 3	1.061 (1.032-1.092)	1.114 (1.082-1.146)	1.043 (1.012-1.075)
Quartile 4	1.177 (1.145-1.211)	1.219 (1.185-1.255)	1.118 (1.080-1.156)
*P* for trend	<0.001	<0.001	<0.001

**Model 1** was unadjusted. **Model 2** was adjusted for age and sex. **Model 3** was adjusted for age, sex, body mass index, total cholesterol, low-density lipoprotein cholesterol, serum creatinine, high sensitivity C-reactive protein, systolic blood pressure, diastolic blood pressure, history of hypertension, history of hyperlipemia, history of chronic kidney disease, hypotensive drugs usage, hypolipidemic drugs usage, income level, education level, physical activity, status of smoking, and status of drinking.

**Analysis 1** excluded participants experienced DM during the first 2 years of follow-up. **Analysis 2** excluded participants experienced atherosclerotic cardiovascular diseases during follow-up. **Analysis 3** excluded all-cause death during follow-up.

## DISCUSSION

Our study confirmed a significant association between the TG/HDL-C ratio and the risk of new-onset DM. Risk of developing DM increased over time according to the baseline TG/HDL-C ratio quartiles. Moreover, the association between TG/HDL-C ratio and DM onset persisted after adjusting for potential confounding variables, with the results remaining consistent across sensitivity analyses.

Similarly, Chen and cols. performed a retrospective cohort study involving 114,787 adults from the Rich Healthcare Group in China. Their results indicated a positive correlation between the TG/HDL-C ratio and DM risk, with a HR of 1.159 (95% CI: 1.104, 1.215). Additionally, they identified a non-linear relation between the TG/HDL-C ratio and DM onset, with an inflection point at a TG/HDL-C ratio of 1.186 (^10^). Several studies explored the association between TG/HDL-C ratio and DM risk across different genders. In this context, Kim and cols. examined data from 80,693 individuals within the Korean NHIS-HEALS cohort database, revealing that an elevated TG/HDL-C ratio was significantly correlated to an increased risk of new-onset DM in both men and women. Specifically, compared with the first tertile, the HRs (95% CIs) for new-onset DM in the second and third tertiles reached 1.17 (1.06-1.30) and 1.47 (1.34-1.62) for men, and 1.20 (1.02-1.42) and 1.52 (1.30-1.78) for women, respectively (^11^). Conversely, Qin and cols. analyzed data from 116,855 individuals and found that a higher TG/HDL-C ratio was significantly associated with DM incidence only in men, with a HR of 1.30 (95% CI: 1.03-1.64) (^12^).

Since type 2 diabetes mellitus (T2DM) is the most common form of DM (^13^,^14^), several studies have investigated the association between TG/HDL-C ratio and the risk of T2DM. Liu and cols. performed a longitudinal retrospective cohort study involving 2,571 participants, finding that higher TG/HDL-C ratio quintiles were associated to increased HRs for T2DM onset compared with the lowest quintile, with HRs of 1.35 (95% CI, 0.85-2.17), 1.31 (0.83-2.06), 1.85 (1.20-2.85), and 2.10 (1.38-3.20), respectively. Additionally, they identified a non-linear relation between the TG/HDL-C ratio and T2DM risk, with the curve slope diminishing after reaching a cutoff point of 2.54 (^15^). Zheng and cols. examined data from 1,460 participants in the Beijing Longitudinal Study, revealing HRs (95% CI) of 1.90 (1.12-3.23), 2.75 (1.58- 4.80), and 2.84 (1.69- 4.77) for TG/HDL-C ratios of 0.87-1.30, 1.31-1.74, and ≥1.75, respectively, in comparison to those with TG/HDL-C ratios below 0.87 (^16^). Wang and cols. analyzed 15,453 Japanese individuals in a cohort study, finding a positive correlation between the baseline TG/HDL-C ratio and T2DM the risk, with a HR of 1.19 (95% CI, 1.09-1.30). Smoothed curve fitting and two-stage linear regression analysis indicated a J-shaped relationship between the baseline TG/HDL-C ratio and T2DM risk. Additionally, a baseline TG/HDL-C ratio exceeding 0.35 was positively correlated with T2DM development, yielding a HR of 1.2 (95% CI, 1.10-1.31) (^17^). Liu and cols. analyzed data from 7,791 participants in the REACTION cohort study, finding a positive association between TG/HDL-C ratio and T2DM risk, with an odds ratio (OR) of 1.49 (95% CI, 1.26-1.78) (^18^).

Prediabetes is identified by blood glucose levels that exceed normal ranges yet fall short of the criteria for diabetes diagnosis, and signifies an elevated risk for DM progression (^19^). Sun and cols. conducted a study involving 15,017 individuals diagnosed with prediabetes. During a median follow-up period of 3.05 years, 1,731 participants (11.46%) were eventually diagnosed with DM. Their results revealed a significant association between TG/HDL-C ratio and DM development in prediabetic individuals (HR = 1.111, 95% CI: 1.061, 1.164). Those with the highest TG/HDL-C ratios exhibited an increased risk of DM progression compared with those with the lowest ratios (HR = 1.397, 95% CI: 1.200, 1.627) (*P* for trend < 0.001) (^20^).

Gestational diabetes mellitus (GDM) constitutes one of the most prevalent complications during pregnancy (^21^). Several studies have specifically focused on the correlation between TG/HDL-C ratio and GDM. Wang conducted an analysis involving 636 women with singleton pregnancies, revealing a GDM prevalence of 17.30% (n = 110). He found that the TG/HDL-C ratios were significantly elevated in the GDM group (1.24 [0.96-1.81]) compared with non-GDM (1.04 [0.80-1.39]) (*P* < 0.01). Additionally, the TG/HDL-C ratio was independently associated with GDM risk (OR = 1.64, *P* = 0.02) (^22^). You and cols. conducted a secondary analysis involving 590 women with singleton pregnancies, indicating a positive association between TG/HDL-C ratio and GDM incidence (OR = 1.77, 95% CI: 1.32-2.38, *P* = 0.0001). Their study also found that the TG/HDL-C ratio serves as a reliable GDM predictor, with an area of 0.7863 (95% CI: 0.7090-0.8637) under the receiver operating characteristic curve. Optimal cut-off value for TG/HDL-C ratio in detecting GDM was 2.2684, yielding a sensitivity of 72.97% and a specificity of 75.05% (^23^).

In conclusion, a higher TG/HDL-C ratio is strongly associated with new-onset DM development in nondiabetic individuals. Our research indicates that managing and maintaining the TG/HDL-C ratio may be helpful in lowering the risk of developing T2DM.

## Data Availability

datasets related to this article will be available upon request to the corresponding author.
